# Transcription Factor SOX5 Promotes the Migration and Invasion of Fibroblast-Like Synoviocytes in Part by Regulating MMP-9 Expression in Collagen-Induced Arthritis

**DOI:** 10.3389/fimmu.2018.00749

**Published:** 2018-04-12

**Authors:** Yumeng Shi, Qin Wu, Wenhua Xuan, Xiaoke Feng, Fang Wang, Betty P. Tsao, Miaojia Zhang, Wenfeng Tan

**Affiliations:** ^1^Department of Rheumatology, The First Affiliated Hospital of Nanjing Medical University, Nanjing, China; ^2^Department of Traditional Chinese Medicine, The First Affiliated Hospital of Nanjing Medical University, Nanjing, China; ^3^Department of Cardiology, The First Affiliated Hospital of Nanjing Medical University, Nanjing, China; ^4^Division of Rheumatology and Immunology, Department of Medicine, Medical University of South Carolina, Charleston, SC, United States

**Keywords:** transcription factor SOX5, matrix metalloproteinase-9, fibroblast-like synoviocytes, migration and invasion, rheumatoid arthritis

## Abstract

**Objectives:**

Fibroblast-like synoviocytes (FLS) exhibit a unique aggressive phenotype in rheumatoid arthritis (RA). Increased FLS migration and subsequent invasion of the extracellular matrix are essential to joint destruction in RA. Our previous research reported that transcription factor SOX5 was highly expressed in RA-FLS. Here, the effects of SOX5 in RA-FLS migration and invasion will be investigated.

**Methods:**

The migration and invasion of RA-FLS were evaluated using a transwell chamber assay. The expression of several potential SOX5-targeted genes, including matrix metalloproteinases (MMP-1, 2, 3 and 9), chemokines (CCL4, CCL2, CCR5 and CCR2), and pro-inflammatory cytokines (TNF-α and IL-6), were examined in RA-FLS using SOX5 gain- and loss-of-function study. The molecular mechanisms of SOX5-mediated MMP-9 expressions were assayed by luciferase reporter gene and chromatin immunoprecipitation (ChIP) studies. The *in vivo* effect of SOX5 on FLS migration and invasion was examined using collagen-induced arthritis (CIA) in DBA/1J mice.

**Results:**

Knockdown SOX5 decreased lamellipodium formation, migration, and invasion of RA-FLS. The expression of MMP-9 was the only gene tested to be concomitantly affected by silencing or overexpressing SOX5. ChIP assay revealed that SOX5 was bound to the MMP-9 promoter in RA-FLS. The overexpression of SOX5 markedly enhanced the MMP-9 promoter activity, and specific deletion of a putative SOX5-binding site in MMP-9 promoter diminished this promoter-driven transcription in FLS. Locally knocked down SOX5 inhibited MMP-9 expression in the joint tissue and reduced pannus migration and invasion into the cartilage in CIA mice.

**Conclusion:**

SOX5 plays a novel role in mediating migration and invasion of FLS in part by regulating MMP-9 expression in RA.

## Introduction

Rheumatoid arthritis (RA) is a common autoimmune disease characterized by chronic inflammation and bone erosion. The synovium is the primary site of inflammatory process, and fibroblast-like synoviocytes (FLS) are considered as the key effector cells in RA (1–3). Compared with normal FLS, RA-FLS is activated and displays a uniquely aggressive and invasive phenotype, including excessive proliferation, resistance to apoptosis, and enhanced migration and invasion (2). The increased migration of FLS to cartilage and bone and subsequent invasion into the extracellular matrix are an essential event in the joint destruction of RA. Inhibition of activated FLS migration and invasion may offer a novel therapeutic strategy for RA (1, 2).

The family of SOX transcription factors contains a conserved high mobility-group (HMG) domain that binds to the minor groove of target DNA elements and regulates transactivation/transrepression of nearby promoters (4). More than 20 different SOX genes have been identified in humans and mice, and they can be further divided into A-H subgroups based on sequence similarity within and outside of the HMG domain. SOX5 belongs to the SoxD group and is expressed in cartilage, heart, brain, and lung (5–8).

The best known function of SOX5 gene is to regulate embryonic development, cell fate determination, and chondrogenesis (4). A recent study suggested that SOX5 was the only SoxD family gene expressed in CD4+ T cells and acted together with c-Maf to induce Th17 cell differentiation (9). It has been shown that SOX5 gene was involved in immune responses since the SOX5 transcript was highly expressed during late stages of B-cell differentiation, which resulted in decreased proliferative capacity of B cells (10).

It was previously reported by our team that a RANKL promoter single-nucleotide polymorphism (SNP) rs7984870 conferred an elevated promoter activity after stimulation, and that it was associated with younger age of RA onset (11). *In silico* analysis demonstrated that the risk allele of this SNP might create a binding site to SOX5 in the RANKL promoter. It was subsequently confirmed that SOX5 was overexpressed in RA-FLS compared with osteoarthritis (OA) FLS and acted as a key regulator for RANKL transcription by directly binding to RANKL promoter in FLS (12). Given the essential role of RANKL in differentiation and bone-resolving activity of osteoclast (13), our data suggested that SOX5 may be involved in the process of bone erosion in RA.

Interestingly, recent studies showed that SOX5 was linked to the migration and invasion of various cancer cells, including hepatocellular carcinoma, melanoma, and prostate cancer (14–16). These findings prompted us to consider whether SOX5 might also regulate tumor-like phenotype of RA-FLS. The current study has shown that SOX5 knockdown reduced the migration and invasion of FLS and matrix metalloproteinase (MMP)-9 expression and also validated that MMP-9 is a target gene of SOX5. These data indicated that SOX5 plays a novel role of SOX5 in mediating migration and invasion of FLS by regulating MMP-9 expression in RA.

## Materials and Methods

### Isolation and Culture of RA-FLS

Synovial tissue samples were collected from RA patients undergoing knee arthroplasty. All diagnosis of RA met the American College of Rheumatology 1987 revised criteria (17). The use of human materials was approved by the Institutional Review Board of the First Affiliated Hospital of Nanjing Medical University, and written informed consent was obtained from all individuals before the operative procedure. FLS were isolated from the synovial tissue samples, and cells were used between the third and eighth passage as described previously (12). The human rheumatoid FLS MH7A cell line used in gene transfection experiments was a generous gift from Dr. Seiichi Tanuma (Tokyo University of Science).

### Lentivirus-shSOX5 and Recombinant Adenovirus SOX5 Expression Vectors Construction and Transfection

For construction of shSOX5, two different small interfering RNA sequences that silenced SOX5 effectively were used in this study (Table S1 in Supplementary Material). The complementary DNA oligonucleotides were subcloned into the hairpin siRNA expression vector pRNAT-U6.1/Neo (Invitrogen). Lentivirus-SOX5 shRNA (LV-shSOX5) and adenovirus SOX5 overexpression vector (Ad-SOX5) were constructed as described previously (12). To ensure specificity of siRNA experiment, a second shRNA sequence targeting SOX5 (LV-shSOX5#2) was designed. All the cloned sequences were confirmed by DNA sequencing.

In knockdown experiments, primary cultured RA-FLS or MH7A cells were seeded on six-well plates at 5 × 10^4^ cells/well and transfected with 2 × 10^6^ TU/mL LV-shSOX5 vectors with 5 μg/mL polybrene (Sigma) for 96 h. Non-targeting shRNAs (mocks) served as negative controls. For SOX5 overexpression experiments, MH7A was transduced with 4 × 10^7^ TU/mL of Ad-SOX5 for 72 h. Control transfectants were obtained by transduction with Ad-enhanced green fluorescent protein (EGFP).

### Cell migration, Invasion, and Wound-Healing Assay

Transwell migration assays were performed by a 24-well Boyden chamber (6.5 mm diameter, 8.0 µm; BD) according to the manufacturer’s instructions. In brief, FLS cells were transfected with LV-shSOX5 for 96 h. The transfected FLS cells were then re-suspended in serum-free media. Approximately 3 × 10^4^ cells/well were seeded on the upper chamber and incubated at 37°C under 5% CO_2_ for 24 h, and the lower chamber was filled with complete media. After 24 h, the residual cells on the upper chamber were gently removed. Migrated cells on the lower side of the membrane were fixed with methanol and stained with 0.1% crystal violet, and counted at 200× magnification at five random fields per well. In the case of *in vitro* invasion assay, similar experiments were performed by these transwell chambers but coated with the Matrigel matrix.

For the wound healing assay, RA-FLS were transfected with LV-shSOX5 and were plated in six-well plates at 70–80% confluence. A linear wound was created by a 200-μl micropipette tip. Detached cells or cell debris were cleaned by two PBS washes, and cells were cultured in serum-free medium. After 24 h of incubation, the migration was quantified by counting the cells that had moved beyond a reference line. Cell migration was determined after 24 h by counting the cells that had moved out of the initial area, and the percentage of cell migration was calculated as [number of migrated cells following treatment/number of migrated cells in control condition] × 100.

### Immunofluorescent Staining for F-Actin

Fibroblast-like synoviocytes cells were transfected with LV-shSOX5 for 96 h and then were re-suspended and seeded into 35-mm confocal dish. After 24 h of incubation, cells were fixed with 4% paraformaldehyde for 20 min and stained with 5 µg/mL FITC-phalloidin (Sigma). Nuclei was co-stained with DAPI (Sigma). Stained cells were examined and photographed by a laser scanning confocal microscope. Lamellipodia and filopodia were classified as previously described (18). Cellular protrusions were scored manually and grouped in following two categories: lamellipodia or filopodia; none of them. The data were shown as [number of cells with lamellipodia or filopodia/total counted cells] × 100 in different conditions. At least 50 cells from 10 random high power fields were counted per condition per experiment.

### Chromatin Immunoprecipitation (ChIP) Assay

Chromatin immunoprecipitation assay was performed using the ChIP A/G kit (Millipore) as described previously (12). In brief, MH7A was cross-linked with 1% formaldehyde for 15 min and 0.125 M glycine for 5 min. Nuclear protein was then isolated from cells. DNA was fragmented into ~200 bp pieces by a Branson 250 sonicator. Each ChIP sample containing 100-µg nucleoprotein was used for the immunoprecipitation reaction with anti-SOX5 (Abcam) and nonspecific IgG, and 10% of the pre-cleared chromatin was set aside as input control. Precipitated genomic DNA was assayed by real-time PCR with the primers (Table S2 in Supplementary Material) encompassing the predicted SOX5-binding site (−1,154/−1,146) on the human MMP-9 gene promoter. Data were normalized to the input control and fold enrichment of the targeted genomic sequences was calculated over IgG.

### Plasmid Construction and Luciferase Reporter Assay

The luciferase constructs containing the 2-kb promoter of MMP-9 gene were previously described (11). The putative SOX5-binding site from −1,300 to −900 was deleted by using the Quick Change II site-directed mutagenesis kit (Stratagene, Cedar Creek, TX, USA) as previously described (11). MH7A cells were seeded on 24-well plates at 2 × 10^5^ cells/well and transfected with Ad-SOX5 or Ad-EGFP for 48 h. After overexpression of SOX5, 1 µg of full-length promoter/luciferase fusion plasmid DNA or the plasmid that deletion of SOX5-binding site was transfected into MH7A by Lipofect-AMINE kit (Invitrogen), respectively. 100 ng of pRL-SV40 control vector (*Renilla* luciferase) was co-transfected as an internal control for transfection efficiency. Luciferase activity in cell lysates was measured after 24 h with a dual luciferase reporter assay (Promega).

### Induction of Collagen-Induced Arthritis (CIA) in DBA/1J Mice and Histological Assessment

The induction of CIA was described previously (12). Briefly, male 6-week-old DBA/1J mice (Shanghai Laboratory Animal Center, Chinese Academy of Science) were injected intradermally at the base of the tail with 200 µg bovine CII (Chondrex) emulsified with complete Freund adjuvant. The animal experiments were performed in accordance with the guidelines approved by Institutional Animal Care and Use Committee of Nanjing Medical University. To evaluate the *in vivo* effect of SOX5 on MMP-9 expression as well as FLS migration and invasion, 1 × 10^7^ TU LV-shSOX5 was intra-articularly injected into the hind ankle at day 1 after the second immunization. Joint tissue was harvested at 23 days after LV-shSOX5 injection.

For histological assessment, the joint tissue was fixed overnight in 4% paraformaldehyde and decalcified using EDTA. The tissue was then embedded in paraffin, sectioned into 1–2 µm, and followed by H&E staining. Rabbit anti-MMP-9 (Santa Cruz) was used as the primary antibodies for immunohistochemistry as we previously reported (12). All slides were coded and submitted for evaluation by investigators blinded to the experimental conditions. The extent of synovitis and pannus formation was determined using a graded scale as described by Tang et al. (19). Briefly, grade 0, no signs of inflammation; grade 1, mild inflammation with hyperplasia of the synovial lining without cartilage destruction; grades 2 through 4, increasing degrees of inflammatory cell infiltrate and cartilage/bone destruction.

### Real-Time PCR and Western Blotting

Levels of gene expression were quantified by SYBR Green real-time PCR by using an ABI Prism 7900 Sequence Detection System. The sequences of the primers were listed in Table S3 in Supplementary Material. Relative expression was normalized to glyceraldehyde-3-phosphate dehydrogenase values by the 2^−ΔΔCt^ method. The following antibodies were used for western blotting: rabbit polyclonal anti-SOX5 antibody (Abcam); goat anti-rabbit MMP-9 antibody (Santa Cruz), rabbit polyclonal anti-β-actin antibody (Cell Signaling Technology).

### Statistical Analysis

For statistical analysis, data were first tested for normality by the Shapiro–Wilk test. For normally distributed variables, pairwise comparisons between two groups were made by un-paired two-tailed Student’s *t*-test. For data that were not normally distributed, statistical analysis was performed by non-parametric Mann–Whitney *U* test. For three groups’ comparison, a one-way ANOVA with the Bonferroni post test was used. Results were reported as mean ± SD. A comparison was considered significant if *p*-values were less than 0.05.

## Results

### SOX5 Knockdown Decreased Migration and Invasion of RA-FLS

**Figure 1:**
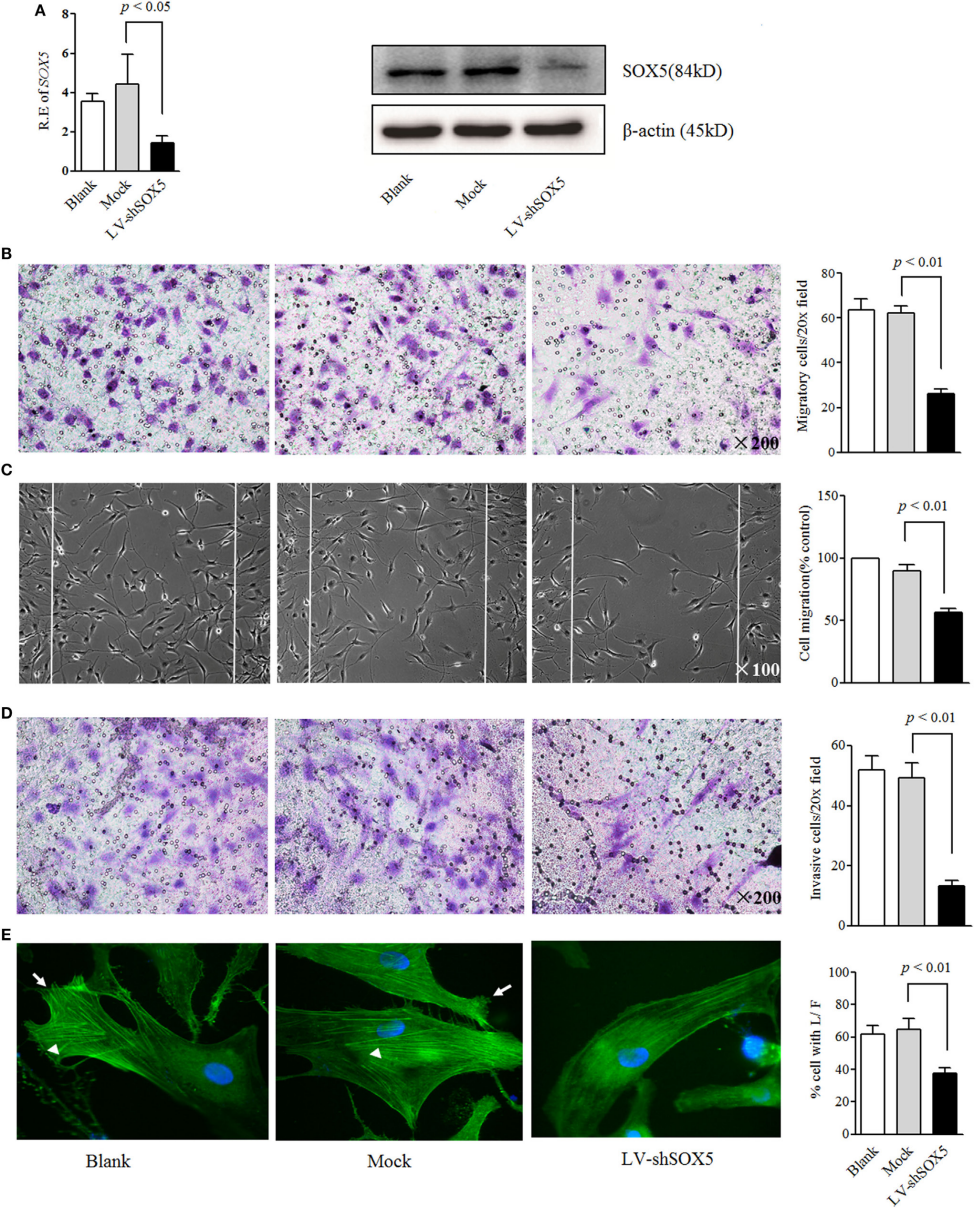
Knockdown *SOX5* inhibits RA-FLS migration and invasion. **(A)** RA-FLS (*n* = 3) were transfected with LV-shSOX5 and mock vector for 96 h. Expression level of *SOX5* mRNA (left) and protein (right) was detected by real-time PCR and western blot. **(B–D)** Following transfection with LV-shSOX5 or Mock for 96 h, fibroblast-like synoviocytes (FLS) subjected to transwell **(B)**, wound healing **(C)**, and transwell chamber invasion assay after 24 h **(D)**. Graphs show the quantitation data derived from the left figure. Data are each representative of three independent experiments. **(E)** Following transfection with LV-shSOX5 or Mock for 96 h, FLS were fixed and stained with FITC-phalloidin. Representative confocal microscopy images of three independent experiments are shown to illustrate stress fibers and appearance of lamellipodia. Stress fibers were highlighted with white triangles and lamellipodia is marked with white arrow. The quantitation data of cellular protrusions is shown in the graph to the right (L/F, lamellipodia and filopodia).

To evaluate the role of SOX5 in RA-FLS invasiveness, LV-shSOX5 was transfected into RA-FLS. This transfection reduced the SOX5 mRNA by 68% (Figure 1A, left) and protein by 60% (Figure 1A right, original images Figure S4) in FLS after 96 h. Correspondingly, as shown in Figure 1B, SOX5-silenced FLS displayed significantly lower cell migration compared with controls in the following 24 h (*p* < 0.01). The wound healing assay confirmed that the migration of LV-shSOX5-transfected FLS was decreased by 40% compared with Mock-treated cells after 24 h (*p* < 0.01) (Figure 1C). Similarly, an *in vitro* invasion assay showed that knockdown of SOX5 expression significantly inhibited invasiveness of RA-FLS (Figure 1D). Together, these data suggested that SOX5 regulated the migration and invasion of RA-FLS.

### SOX5 Knockdown Affected the Cytoskeletal Reorganization in RA-FLS

Cell migration and invasion are commonly accompanied by dynamic reorganization of the actin cytoskeleton. To further confirm the effects of SOX5 on FLS migration and invasion, the rearrangements of the actin cytoskeleton in RA-FLS in response to SOX5 knockdown was examined using F-actin staining. As shown in confocal images, focal adhesion-anchored stress fibers, lamellipodia, and filopodia were clearly visible in Mock-treated RA-FLS and blank group after being seeded onto culture dish for 24 h (Figure 1E, left and middle). Knockdown of SOX5 markedly decreased the appearance of stress fibers and prevented the formation of lamellipodia and filopodia in RA-FLS (Figure 1E, right), implying that SOX5 was essential for F-actin remodeling that required for efficient cell migration.

### SOX5 Mediated the Migration and Invasion of RA-FLS by Regulating MMP-9 Expression

**Figure 2:**
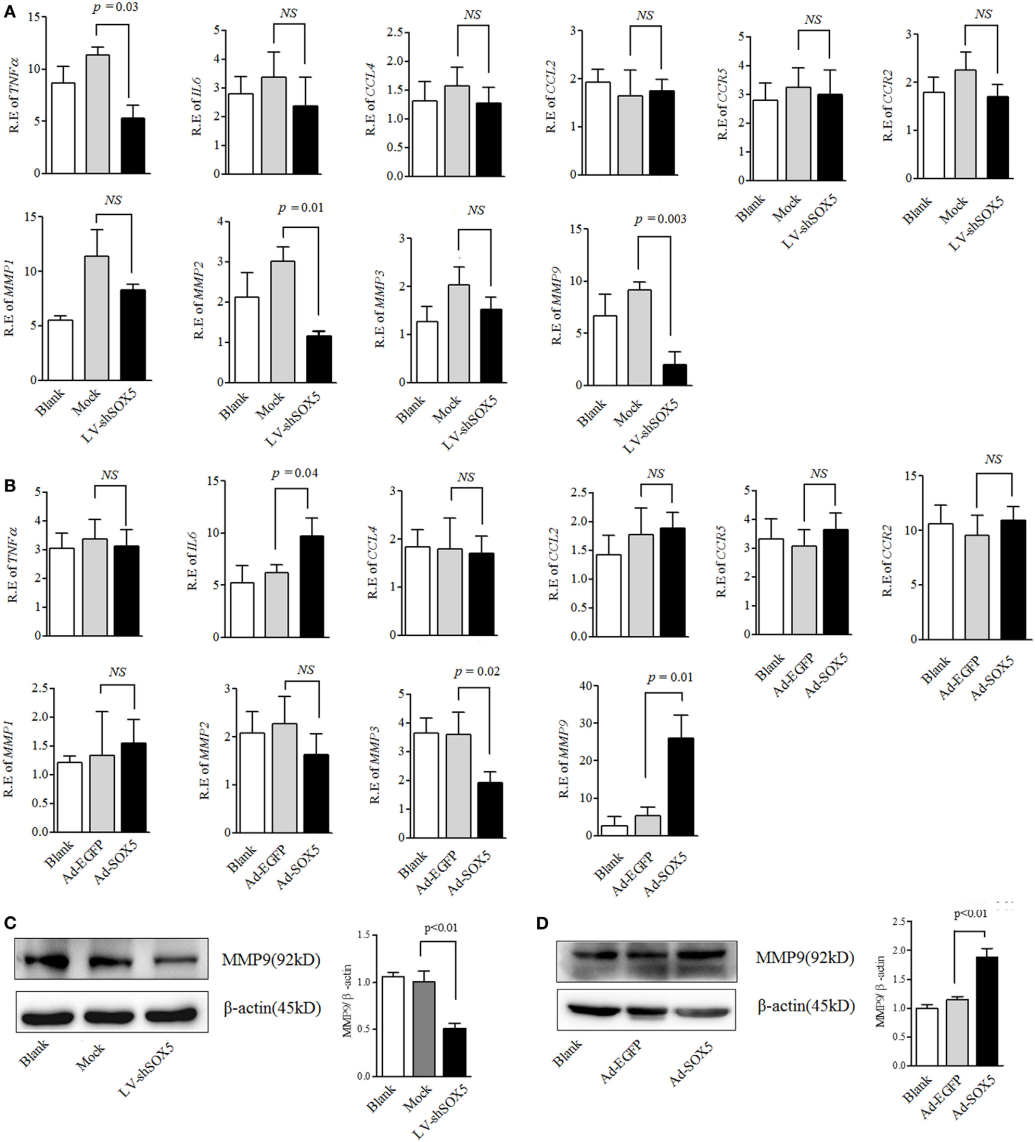
SOX5-mediated RA-FLS migration and invasion by regulating MMP-9 expression. **(A)** Following knockdown SOX5 in fibroblast-like synoviocytes (FLS) for 96 h by transfection with LV-shSOX5 or Mock vector, expression of TNF-α, IL-6, CCL4, CCL2, matrix metalloproteinase (MMP)-1, 2, 3, and 9 were detected by real-time PCR. **(B)** Following overexpressing SOX5 in FLS for 72 h by transfection with Ad-SOX5 or Ad-EGFP, expression of TNF-α, IL-6, CCL4, CCL2, MMP-1, 2, 3, and 9 were detected by real-time PCR. **(C,D)** Protein levels of MMP-9 in SOX5 knockdown **(C)** and overexpressing **(D)** FLS. Data are each representative of three independent experiments in different FLS lines (*n* = 3).

Expressions of MMPs, chemokines, and pro-inflammatory cytokines by RA-FLS are necessary for their migration and invasion (2, 20). We next investigated whether SOX5 affected RA-FLS migration and invasion through MMP-1, 2, 3, 9, TNF-α, IL-6, CCL4 (MIP-1b) and its receptor CCR5 or CCL2 (MCP-1) and its receptor CCR2. As shown in Figure 2A, no significant change of other tested genes was observed at 96 h post transfection even though SOX5 knockdown markedly decreased expression of TNF-α (*p* = 0.03), MMP-2 (*p* = 0.01), and MMP-9 (*p* = 0.003) in RA-FLS. Overexpression of SOX5 by transfected with Ad-SOX5 for 72 h resulted in dramatic increase in the expression of IL-6 (*p* = 0.04), MMP-3 (*p* = 0.02), and MMP-9 (*p* = 0.01) (Figure 2B).

**Figure 3:**
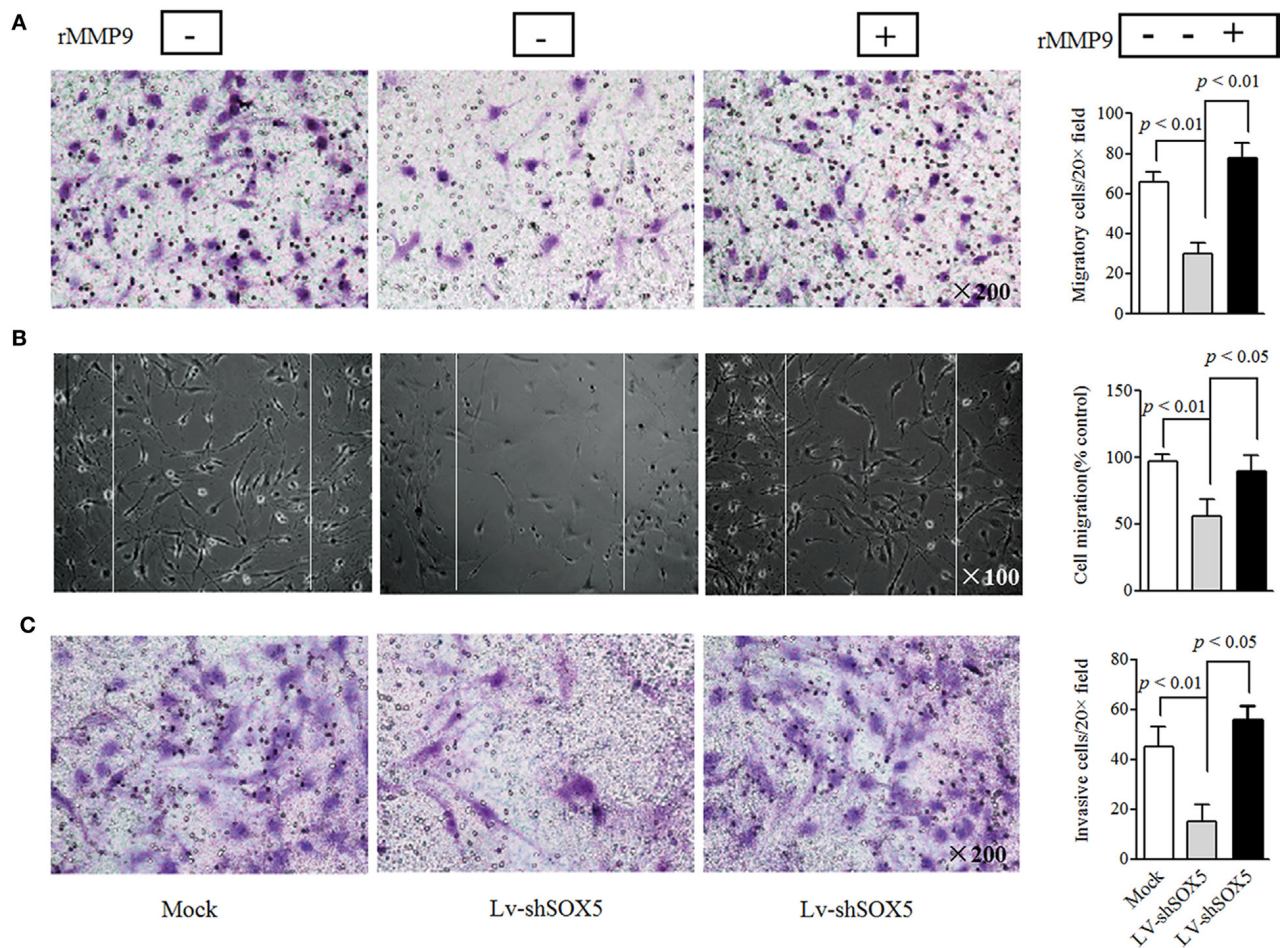
Recombinant matrix metalloproteinase (MMP)-9 rescues the inhibitory effect of SOX5 knockdown on fibroblast-like synoviocytes (FLS) migration and invasion. Following knockdown SOX5 in FLS for 96 h, FLS with or without recombinant MMP-9 (50 ng/mL) were subjected to transwell **(A)**, wound healing **(B)**, and transwell chamber invasion assay **(C)** after 24 h. Graphs show the quantitation data. Data are each representative of three independent experiments.

In the above gain- and loss-of-function study, only MMP-9 was concomitantly affected by silencing or overexpressing SOX5, suggesting that SOX5 specifically regulates MMP-9 expression. Similarly, western blotting revealed a decreased or increased protein level of MMP-9 in RA-FLS in response to silencing (Figure 2C, original images Figure S4) or overexpressing SOX5 (Figure 2D, original images Figure S5). We then validated the effect of MMP-9 on RA-FLS phenotype by transfection of MMP-9-siRNA (Thermo Fisher Scientific, #AM16708) into FLS. As expected, FLS showed the decreased migration and invasion abilities after MMP-9 knockdown (Figure S1 in Supplementary Material). To confirm that MMP-9 is responsible for SOX5-mediated migration and invasion of RA-FLS, recombinant MMP-9 protein (r-MMP-9, 50 ng/ml) was added into LV-shSOX5 transfected RA-FLS. As shown in Figures 3A–C, r-MMP-9 could rescue the inhibitory effect of SOX5 knockdown on migration and invasion in RA-FLS. To confirm the specificity of observed biological effects after SOX5 knockdown on FLS, we designed a second shRNA sequence targeting SOX5 (LV-shSOX5#2), which was as effective as the first shRNA on knockdown SOX5 gene expression (data not shown). Similar to the first LV-shSOX5, LV-shSOX5#2 transfected RA-FLS showed the decreased capacity of cell migration and invasion and the decreased ability can be in part reversed by r-MMP-9 (Figure S2 in Supplementary Material).

### SOX5 Regulated MMP-9 Transcription

**Figure 4:**
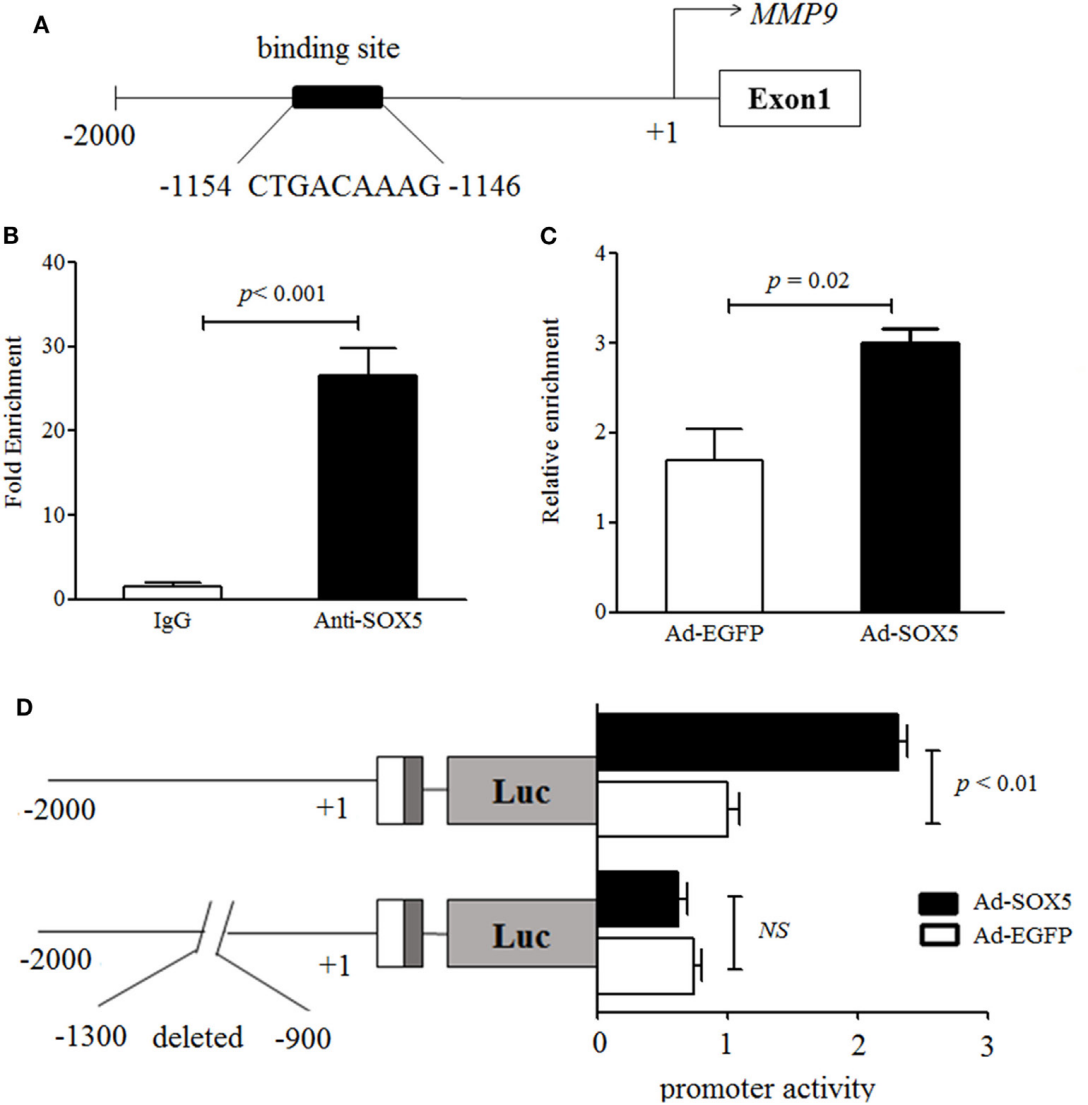
SOX5 promotes matrix metalloproteinase (MMP)-9 transcription *via* binding to the promoter region. **(A)** Schematic diagram of one putative SOX5-binding sites (−1,154~−1,146) in MMP-9 promoter region. **(B)** MH7A cell lysates were immunoprecipitated by Anti-SOX5 antibody or rabbit IgG as control. qPCR analyses the enrichment of MMP-9-specific DNA in MH7A cells. **(C)** MH7A cells were transfected with Ad-SOX5 or Ad-EGFP for 72 h, and then harvested for chromatin immunoprecipitation assay. The cell lysates were immunoprecipitated by SOX5 antibody. qPCR analyses the enrichment of MMP-9-specific DNA in MH7A cells. **(D)** Truncation analysis of the *MMP-9* reporters. Two kinds of reporters, including the full 2-kb MMP-9 promoter and deletion putative SOX5-binding site from −1,300 to −900 in promoter, were analyzed for their activity. MH7A cells were transfected with Ad-SOX5 or Ad-EGFP for 48 h, then transfected with these two kinds of reporters for 24 h, respectively. Data are each representative of three independent experiments.

To further explore the relationship between SOX5 and MMP-9, it was investigated whether MMP-9 is a direct target gene of transcriptional factor SOX5. A direct target gene should contain one or more functional SOX5-binding site (s) in the promoter. At least four putative SOX5-binding sites have been identified in the 2-kb promoter of MMP-9 by an online software (http://jaspar.genereg.net/) (Figure S3 in Supplementary Material). One putative binding site located at −1,154 to −1,146 with the highest predicted score was selected for our investigation (Figure 4A). The ChIP assay showed that a higher amount of chromatin containing MMP-9 promoter region was immunoprecipitated by anti-SOX5 antibody compared to that obtained by control IgG in RA-FLS (Figure 4B). Furthermore, overexpressing SOX5 for 72 h in MH7A resulted in approximate twofold increase of the enrichment of MMP-9-specific DNA in anti-SOX5 immunoprecipitate compared to that from none-SOX5 transfected MH7A (Figure 4C).

To confirm the regulatory role of SOX5 in the MMP-9 promoter, two luciferase reporter constructs were generated, and transfected into MH7A, respectively. One construct was driven by full 2-kb promoter fragments extending from −2,000 to +1 and another was driven by deletion putative SOX5-binding site from −1,300 to −900 of the proximal promoter. Overexpression of SOX5 increased MMP-9 promoter activity by ~2.5-fold compared to control (EGFP) in MH7A (Figure 4D, above). However, no enhanced MMP-9 promoter activity was observed in cells transfected with plasmids carrying a deletion of the putative SOX5-binding site (Figure 4D, bottom). Collectively, these results indicated that MMP-9 contains at least one functional SOX5-binding site in its promoter and represents a direct SOX5 target gene. Therefore, the binding of SOX5 to the MMP-9 promoter plays a key role in regulating MMP-9 transcription.

### SOX5 Knockdown Affected p38-MAPK Pathway Activation in RA-FLS

**Figure 5:**
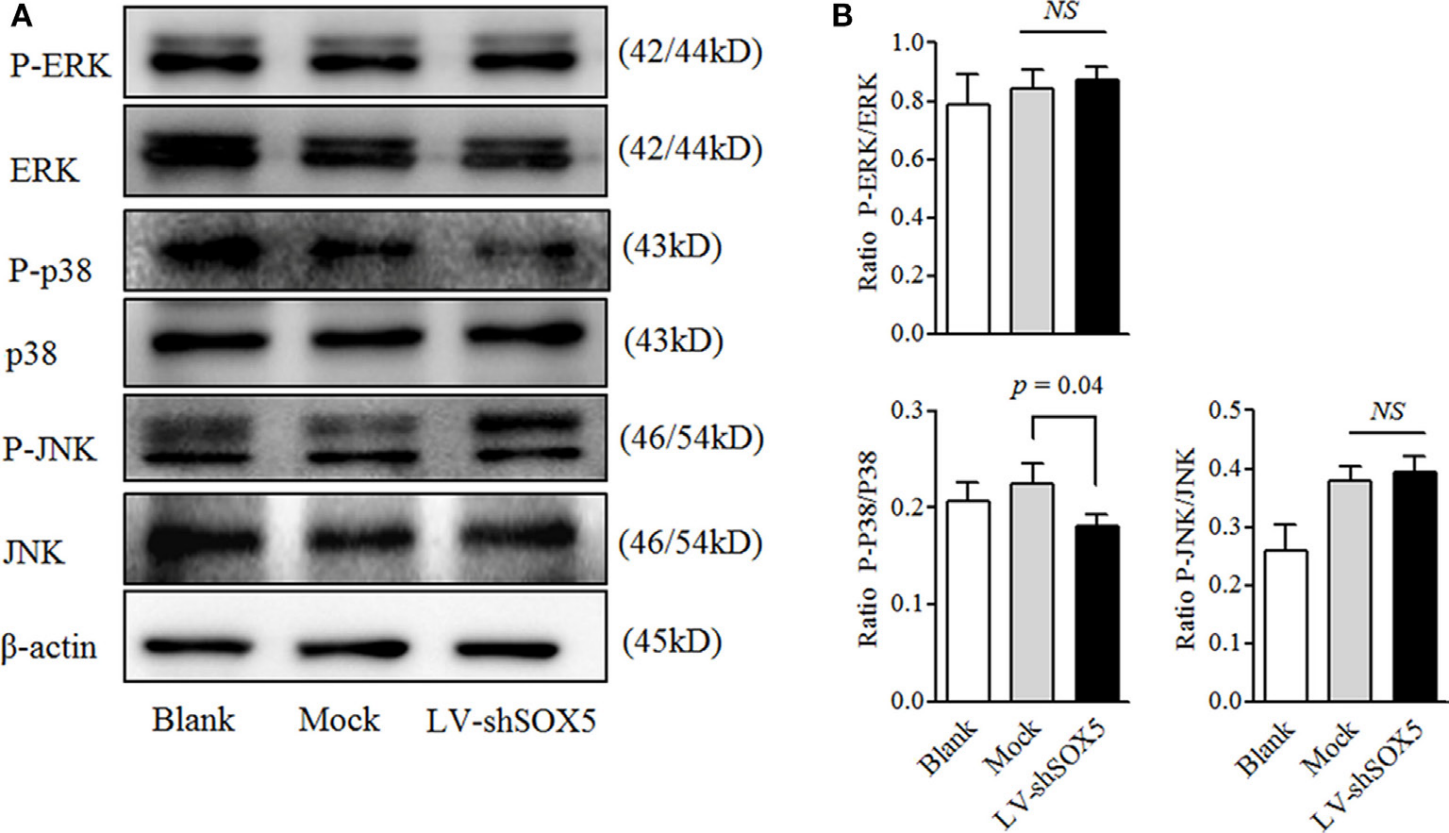
Knockdown SOX5 inhibits p38-MAPK phosphorylation in RA-FLS. **(A)** Following transfected with LV-shSOX5 or Mock for 96 h, cell lysates were subjected to western blotting analysis for the phosphorylation of ERK, p38, and JNK. The results are representative of three similar, independent experiments. **(B)** Graphs show the quantitation data derived from the left figure. Data are each representative of three independent experiments.

MAPK signaling pathways are classical ones for MMP-9 activation and play an important role in regulating cell migration (21). As shown in Figure 5, among three tested MAPK pathways (JNK, ERK, and p38), only the activation of p38 was significantly decreased in MH7A after transfection with LV-shSOX5 for 96 h, as compared with Mock-treated cells (Figures 5A,B, original images Figures S6,7). Our data suggested that p38-MAPK pathway is involved in SOX5-mediated MMP-9 expression in RA-FLS.

### Locally Knockdown SOX5 in CIA Mice Inhibited FLS Migration and Invasion Likely by Inhibiting MMP-9 Expression

**Figure 6:**
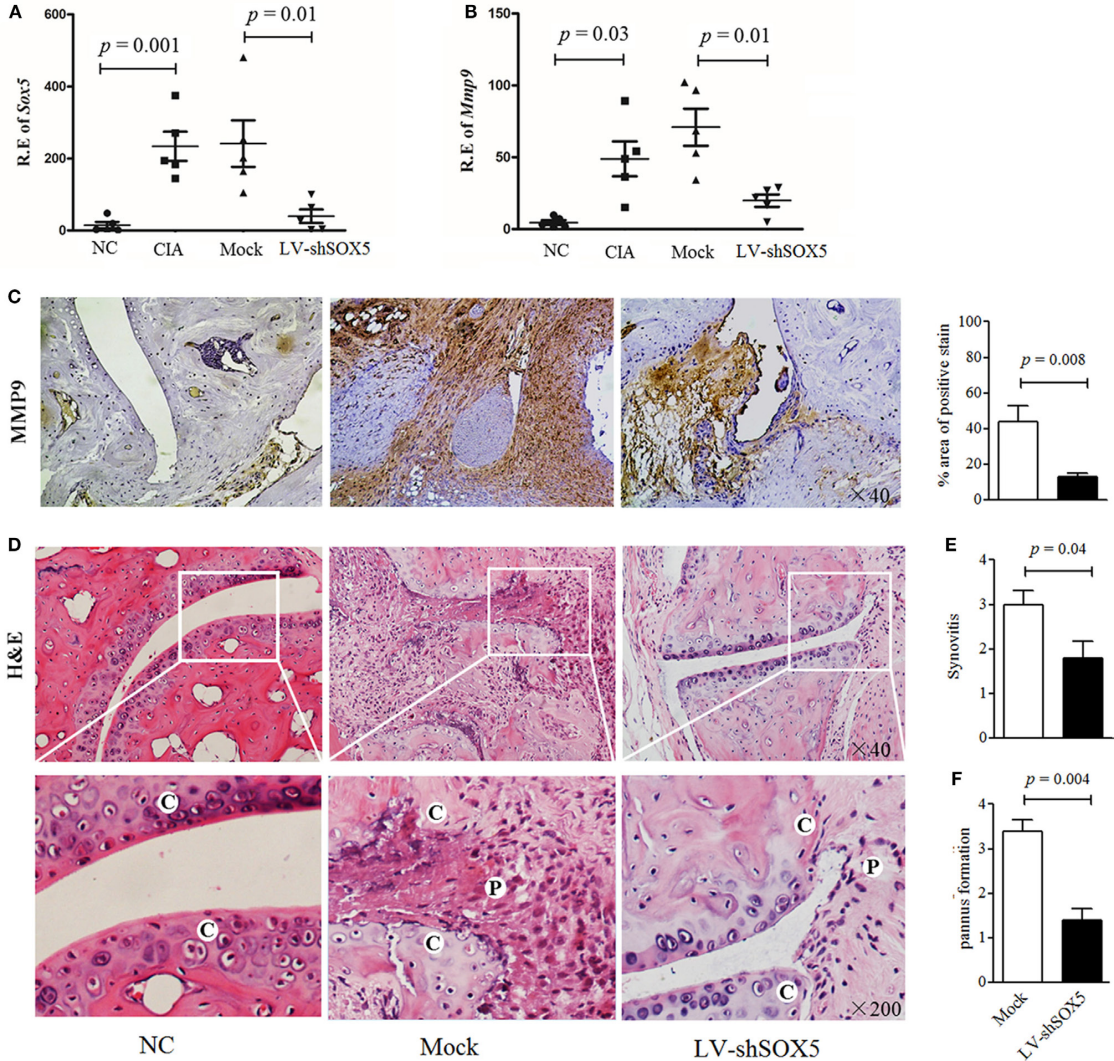
Local suppression of Sox5 in joints of collagen-induced arthritis (CIA) mice inhibited fibroblast-like synoviocytes (FLS) migration and invasion by decreasing Mmp-9 expression. **(A,B)** Relative expression of Sox5 **(A)** and Mmp-9 mRNA **(B)** in arthritic joint from NC, CIA, mock shRNA and LV-shSox5 treated CIA mice at day 24 after the second immunization (*n* = 5, per group). **(C)** Immunohistochemical detection of Mmp-9 positive cell expression in synovium samples from CIA mice after treatment with LV-shSox5 or Mock vector. **(D)** H&E histological analysis of representative ankle sections from NC and CIA mice treated with LV-shSox5 or Mock vector at day 24 after the second immunization. *NC*, normal control, *C*, cartilage, *P*, pannus. **(E,F)** Quantitation of synovitis **(E)** and pannus formation **(F)** (*n* = 5). Synovitis and pannus formation (cartilage–pannus interface) were scored from 0 to 4.

Our previous data showed that local knockdown of SOX5 expression decreased arthritis incidence almost by 50%, and markedly inhibited synovitis, synovial hyperplasia, and bone erosion in a murine CIA model (12). To elucidate the *in vivo* effect of SOX5 on MMP-9 expression and FLS migration and invasion, local SOX5 expression was knockdown by injecting LV-shSOX5 intra-articularly into the hind ankle at day 1 after the second immunization in CIA mice (*n* = 5). As expected, this injection decreased SOX5 mRNA in the joint tissue more effectively than mock shRNA administration (Figure 6A, *p* = 0.001). Accordingly, MMP-9 mRNA expression was markedly decreased in mouse arthritic joint administered with LV-shSOX5 (Figure 6B, *p* = 0.01). Similarly, immunohistochemistry staining showed that MMP-9 protein expression was diminished in synovial samples from LV-shSOX5-treated CIA mice compared to those from mock-treated mice (Figure 6C).

Consistently, mice locally treated with LV-shSOX5 exhibited significantly lower levels of synovitis (Figures 6D,E), cartilage erosion and pannus formation adjacent to cartilage tissue (Figures 6D,F) than control, indicating that knockdown SOX5 expression inhibited pannus migration and invasion into the cartilage during CIA development.

In summary, our findings suggested that SOX5 regulates MMP-9 expression, prompting RA-FLS migration and invasion into the cartilage and bone during RA development.

## Discussion

SOX5 is traditionally known as a key regulator for embryonic development and determination of cell fate. This gene attracted our attention, as our previous results identified that a novel RA-associated SNP rs7984870 could create a binding site for transcriptional factor SOX5 in *RANKL* promoter (11). Later more of our results for the first time showed the significant overexpression of SOX5 in RA compared to OA FLS, and confirmed that SOX5 played a key role in regulating RANKL expression in RA-FLS (12). We now extended our previous study and revealed a new function of SOX5 in promoting migration and invasion of RA-FLS *via* modulation MMP-9 expression. These data suggested that SOX5 plays a crucial role in RA progression.

RA-FLS possess a tumor-like migration and invasion behavior. Once these cells arrive at the bone, they can deeply invade into the extracellular matrix and then destroy bone and cartilage by releasing matrix degrading enzymes, such as MMPs and cathepsins (1, 2). RA-FLS, but not normal FLS, could migrate and invade into co-implanted human cartilage in the severe combined immune deficient mice (22, 23), indicating the intrinsic properties of RA-FLS contribute to this invasive behavior. However, to date, the underlying mechanisms are poorly understood.

The current *in vitro* assays revealed that SOX5 was associated with the migration and invasion of RA-FLS. This action of SOX5 is likely *via* rearrangement of actins, as protrusion of lamellipodia initiates the migration of various cell types including fibroblasts (24). Moreover, pannus migration and invasion into the cartilage *in vivo* CIA model was greatly reduced after locally SOX5 silencing. These data revealed a crucial role for SOX5 in promoting FLS migration and invasion both *in vitro* and *in vivo*.

Another main finding of this study is that SOX5 regulates MMP-9 expression in RA-FLS. The gain- and loss-of-function studies confirmed that the effect of SOX5 on FLS migration and invasion is likely regulated by MMP-9. MMP-9 has been found in RA synovial fluid and FLS (20, 25, 26). In addition to degrading the matrix in RA, MMP-9 regulates angiogenesis and pannus formation in RA (27). MMP-9, produced by RA-FLS but not normal or OA FLS, contributes to RA-FLS survival, inflammation and cartilage degradation (28, 29). Our previous study has shown that inflammatory cytokines including IL-6 and TNF-α are the potent drivers for SOX5 expression in RA-FLS (12). Based on these studies, it was proposed that the increased SOX5 in RA inflammatory condition exerted its pro-migration and pro-invasion function for FLS, at least in part, by promoting MMP-9 expression.

A variety of transcription factors participate in regulating MMPs expression. Pro-inflammatory cytokines, such as IL-1 or TNF-α, induce the MMPs expression including MMP-9 *via* binding to activator protein-1-binding site in the MMPs promoter (2, 30). The MMP promoters also contain NF-κB-like binding sites that are involved in promoting MMP production in cytokine stimulated FLS (2). Our current study identified one SOX5-binding site within the proximal MMP-9 promoter by ChIP assay. *Via* binding to this site, SOX5 enhanced MMP-9 expression in RA-FLS. This was further confirmed by the fact that even overexpression of SOX5 failed to transactivate MMP-9 after deletion of this SOX5-binding element in MMP-9 promoter. This evidence suggests that MMP-9 is a target gene of transcriptional factor SOX5 in RA-FLS. Indeed, more than one SOX5-binding sites have been identified in MMP-9 promoter by our bioinformatics analysis. But to identify the critical core binding site of SOX5 is beyond the scope of the current study.

Our previous study has proved that SOX5 participate in regulating RANKL expression in RA-FLS (12). Here, the current data demonstrated that SOX5 plays an important role in MMP-9-mediated FLS aggressive behavior. Although multiple and complex processes are involved in bone erosion of RA, FLS and osteoclast are two key effector cells responsible for joint destruction. The direct effect on FLS aggressive behavior and on osteoclastogenesis-related RANKL gene expression highlights a critical role of SOX5 in RA progression. As a transcriptional factor, SOX5 can target multiple genes, such as COL2A1 (31), SPARC (32), TWIST (14), and RORγt (9). Those target genes exert their functions into chondrogenesis, tumor progression and Th17 cell differentiation. It is possible that MMP-9 is only one of the downstream genes of SOX5. In addition to MMP-9, other gene or pathways targeted by SOX5 might contribute to the invasive phenotype in RA-FLS. Further studies are needed to explore the effect of other mechanism in which SOX5 is mediated in RA pathogenesis.

In conclusion, our current research is the first attempt to identify SOX5 as a regulator to promote MMP-9 expression and to potentiate the migration and invasion activity in RA-FLS. Our data highlight the relevance of SOX5 as a potential novel therapeutic target in RA.

## Ethics Statement

This study was carried out in accordance with the recommendations of the National Institute of Health Guide for the Care and Use of Laboratory Animals and Animal Care and Use Committee at the Nanjing medical University. The protocol was approved by the Animal Care and Use Committee at the Nanjing Medical University (Permit Number: IACUC-2014080103).

## Author Contributions

WT, WX, BT, and YS were involved in the design of the study. QW, WX, XF, YS, and FW were involved in the conduct of the study. WT and XF undertook analysis, and all authors were involved in interpretation of the data. WT, YS, and BT prepared the manuscript, and all authors were involved in the review and approval of the final manuscript.

## Conflict of Interest Statement

The authors declare that the research was conducted in the absence of any commercial or financial relationships that could be construed as a potential conflict of interest.

## Supplementary Material

Figure S1 Knockdown MMP-9 inhibits RA FLS migration and invasion. Following transfected with LV-shSOX5 or Mock for 24h, FLS (*n = 3*) subjected to transwell (above), and transwell chamber invasion assay after 24 h (below). Graphs show the quantitation data derived from the left figure. Data are each representative of three independent experiments.

Figure S2 Recombinant MMP-9 rescues the inhibitory effect of LV-shSOX5#2 on FLS migration and invasion. Following knockdown SOX5 with LV-shSOX5#2 treated in FLS for 96 h, FLS with or without recombinant MMP-9 (50 ng/mL) were subjected to transwell **(A)**, and transwell chamber invasion assay **(C)** after 24 h. MH7A cell was used in wound healing test **(B)**. Graphs show the quantitation data. Data are each representative of three independent experiments.

Figure S3 DNA sequence of 2-kb MMP-9 promoter. Four putative SOX5-binding sites have been identified in the 2-kb promoter of MMP-9 by an online software (http://jaspar.genereg.net/). One putative binding site located at −1154 to −1146 with the highest predicted score was marked with red. The deleted putative SOX5 binding site from −1300 to −900 of the proximal promoter was marked with italic.

Figure S4 Original images of Figures 1A,2C.

Figure S5 Original images of Figure 2D.

Figure S6 Original images of Figure 5A-1.

Figure S7 Original images of Figure 5A-2.
