# Supplementary figures and images for: Transcription Factor SOX5 Promotes the Migration and Invasion of Fibroblast-Like Synoviocytes in Part by Regulating MMP-9 Expression in Collagen-Induced Arthritis
Source: Front Immunol. 2018 Apr 12;9:749. doi: 10.3389/fimmu.2018.00749 (PMC5906798; doi:10.3389/fimmu.2018.00749)

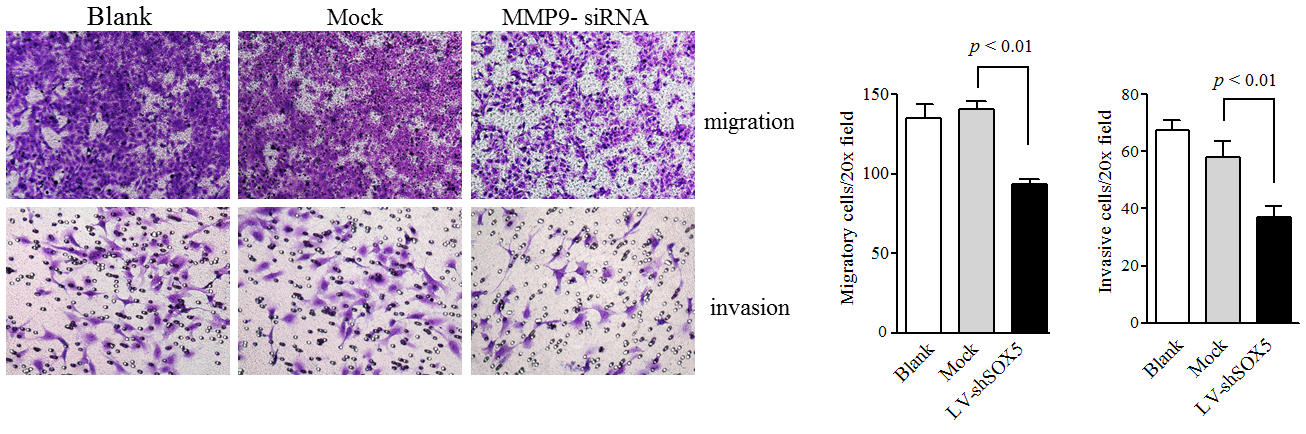

Supplement: Figure S1 — Knockdown MMP-9 inhibits RA FLS migration and invasion. Following transfected with LV-shSOX5 or Mock for 24h, FLS (n = 3) subjected to transwell (above), and transwell chamber invasion assay after 24 h (below). Graphs show the quantitation data derived from the left figure. Data are each representative of three independent experiments. [file Image_1.TIF]

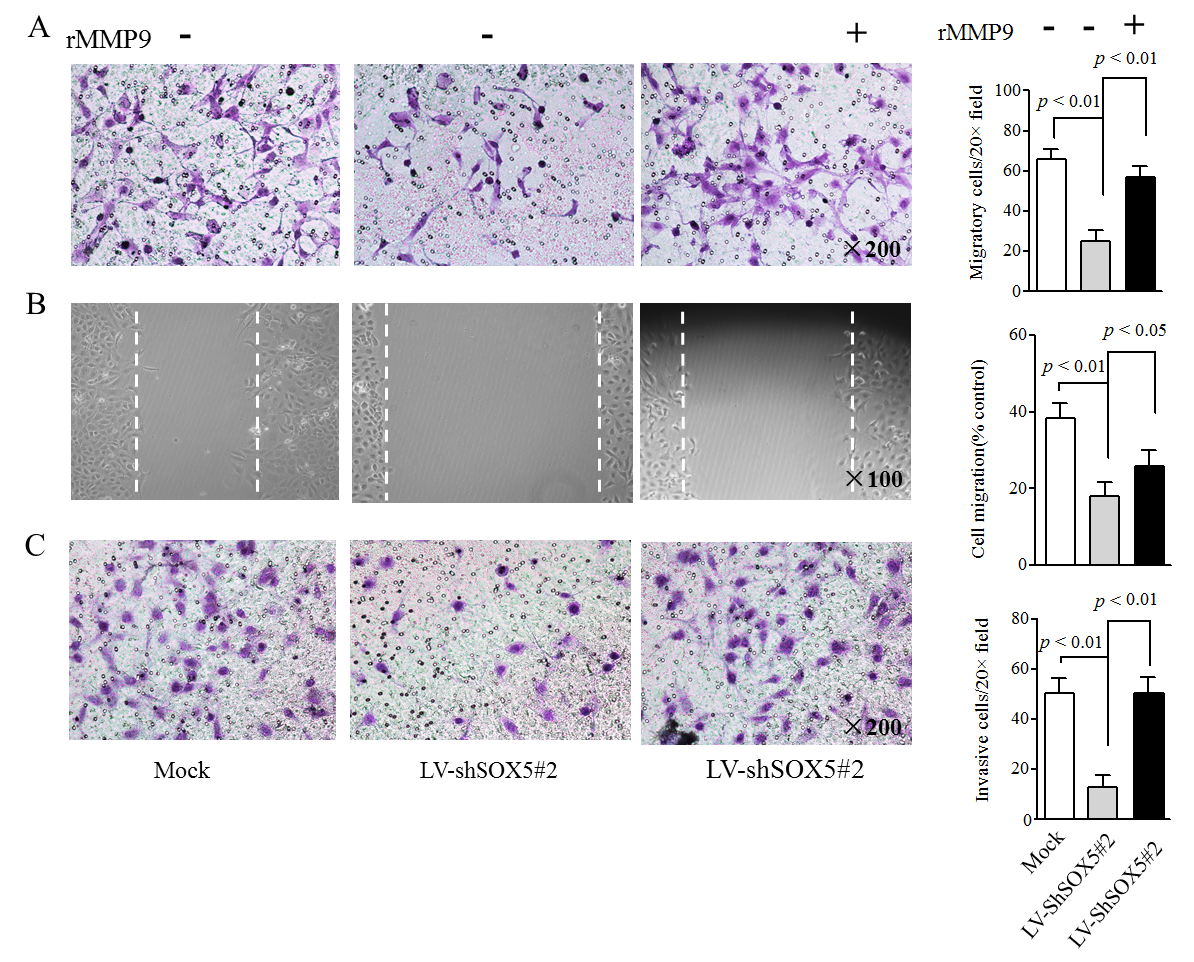

Supplement: Figure S2 — Recombinant MMP-9 rescues the inhibitory effect of LV-shSOX5#2 on FLS migration and invasion. Following knockdown SOX5 with LV-shSOX5#2 treated in FLS for 96 h, FLS with or without recombinant MMP-9 (50 ng/mL) were subjected to transwell (A), and transwell chamber invasion assay (C) after 24 h. MH7A cell was used in wound healing test (B). Graphs show the quantitation data. Data are each representative of three independent experiments. [file Image_2.TIF]

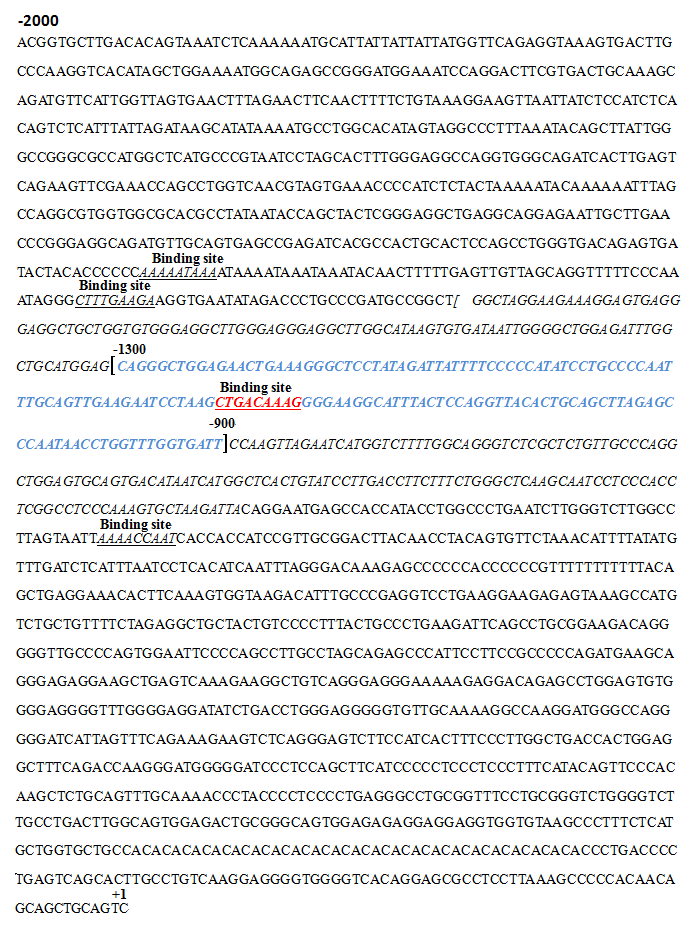

Supplement: Figure S3 — DNA sequence of 2-kb MMP-9 promoter. Four putative SOX5-binding sites have been identified in the 2-kb promoter of MMP-9 by an online software (http://jaspar.genereg.net/). One putative binding site located at −1154 to −1146 with the highest predicted score was marked with red. The deleted putative SOX5 binding site from −1300 to −900 of the proximal promoter was marked with italic. [file Image_3.TIF]

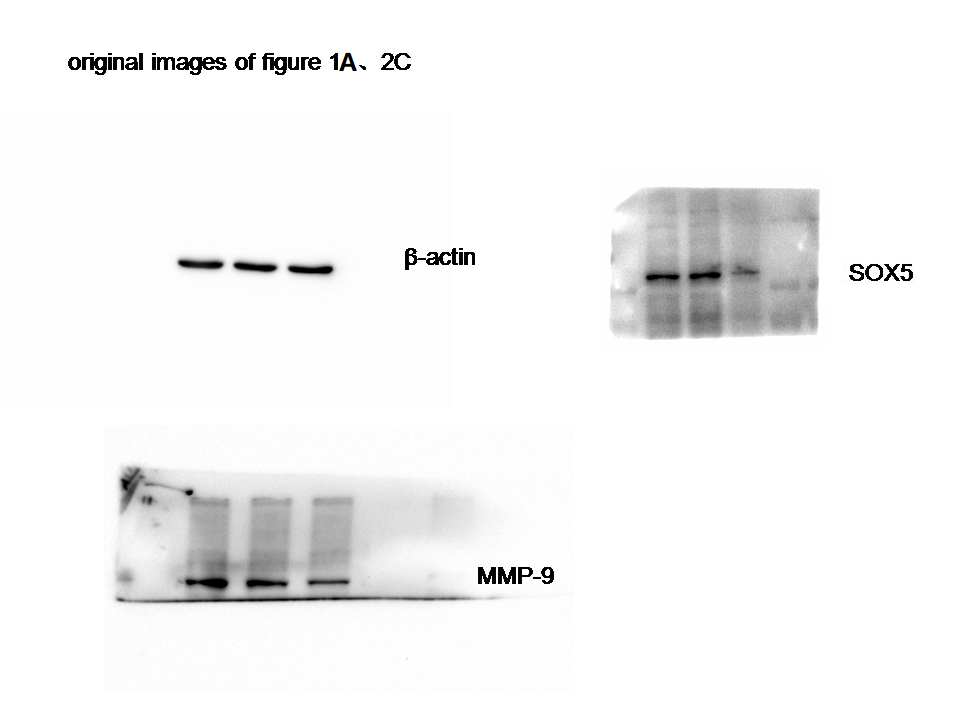

Supplement: Figure S4 — Original images of Figures 1A,2C. [file Image_4.TIF]

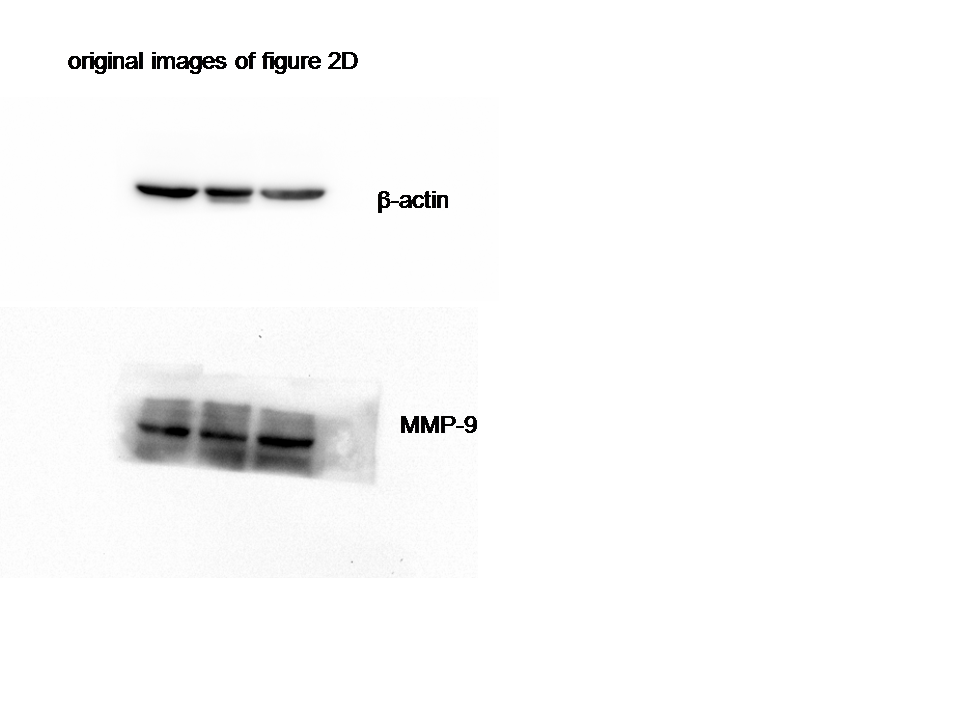

Supplement: Figure S5 — Original images of Figure 2D. [file Image_5.TIF]

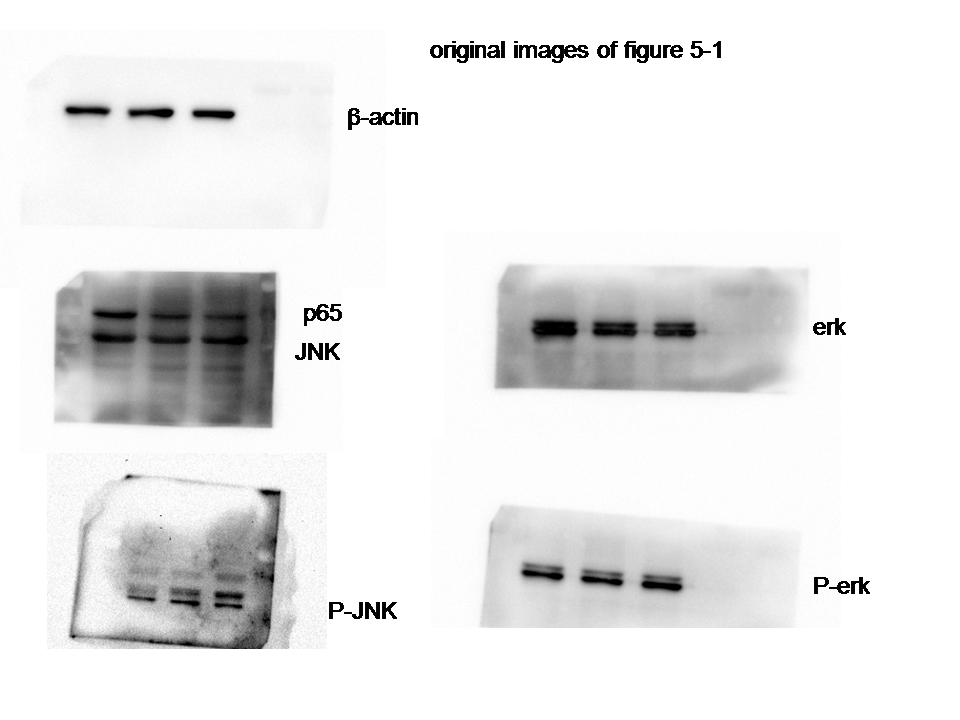

Supplement: Figure S6 — Original images of Figure 5A-1. [file Image_6.TIF]

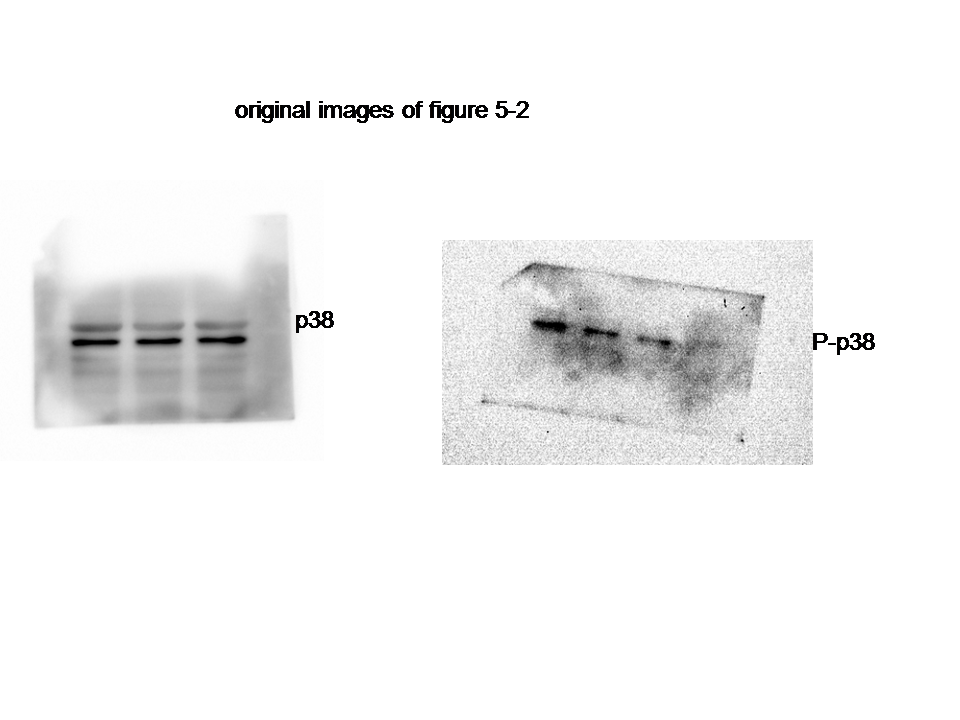

Supplement: Figure S7 — Original images of Figure 5A-2. [file Image_7.TIF]
