# Supplementary material for: Transcription Factor SOX5 Promotes the Migration and Invasion of Fibroblast-Like Synoviocytes in Part by Regulating MMP-9 Expression in Collagen-Induced Arthritis
Source: Front Immunol. 2018 Apr 12;9:749. doi: 10.3389/fimmu.2018.00749 (PMC5906798; doi:10.3389/fimmu.2018.00749)
Supplement: Supplementary file 8 [file Table_1.DOC]

**Table S1. siRNA sequences**

| Species | Strand | Sequences |
| --- | --- | --- |
| Mouse *SOX5* | Sense  Antisense | 5’-GATCCCGCTCCATACAACTCATCTATTGATATCCGTAGATGAGTTGTATGGAGCTTTTTTCCAAA-3’  5’- AGCTTTTGGAAAAAAGCTCCATACAACTCATCTACGGATATCAATAGATGAGTTGTATGGAGCGG-3’ |
| Human *SOX5* | Sense  Antisense | 5’- GATCCCGAGCACTTACGGTGTGAAATTGATATCCGTTTCACACCGTAAGTGCTCTTTTTTCCAAA-3’  5’- AGCTTTTGGAAAAAAGAGCACTTACGGTGTGAAACGGATATCAATTTCACACCGTAAGTGCTCGG-3’ |
