# Supplementary material for: Transcription Factor SOX5 Promotes the Migration and Invasion of Fibroblast-Like Synoviocytes in Part by Regulating MMP-9 Expression in Collagen-Induced Arthritis
Source: Front Immunol. 2018 Apr 12;9:749. doi: 10.3389/fimmu.2018.00749 (PMC5906798; doi:10.3389/fimmu.2018.00749)
Supplement: Supplementary file 9 [file Table_2.DOC]

**Table S2. ChIP-PCR primer**

| Genes  (Human) | Forward | Reverse |
| --- | --- | --- |
| *MMP9* | TAAGCTGACAAAGGGGAAGG | CCTGCCAAAAGACCATGATT |
