# Supplementary material for: Transcription Factor SOX5 Promotes the Migration and Invasion of Fibroblast-Like Synoviocytes in Part by Regulating MMP-9 Expression in Collagen-Induced Arthritis
Source: Front Immunol. 2018 Apr 12;9:749. doi: 10.3389/fimmu.2018.00749 (PMC5906798; doi:10.3389/fimmu.2018.00749)
Supplement: Supplementary file 10 [file Table_3.DOC]

Table S3. Gene-specific primers

| Genes  ( Human) | Forward | Reverse |
| --- | --- | --- |
| *ACTB* | CCACACTGTGCCCATCTACG | AGGATCTTCATGAGGTAGTCAGTCAG |
| *SOX5* | CAGCCAGAGTTAGCACAATAGG | CTGTTGTTCCCGTCGGAGTT |
| *MMP1* | AAAATTACACGCCAGATTTGCC | GGTGTGACATTACTCCAGAGTTG |
| *MMP2* | GAGGAGCAGTTACGGTCTGTG | TCCTTTCCTTAGCTGACACTTGT |
| *MMP3* | AGTCTTCCAATCCTACTGTTGCT | TCCCCGTCACCTCCAATCC |
| *MMP9*  *TNFα*  *IL-6*  *CCL4*  *CCL2*  *CCR5*  *CCR2* | TGTACCGCTATGGTTACACTCG  GGAGAAGGGTGACCGACT C GGTACATCCTCGACGGCAT CTA  CTTTTCTTACACCGCGAGGA  CAGCCAGATGCAATCAATGCC  TGTGGGCAACATGCTGGTCATC  TGACAGGCACAGATGAATGG | GGCAGGGACAGTTGCTTCT  CTGCCCAGACTCGGCAA  GTGCCTCTTTGCTGCTTTCAC  GCTTGCTTCTTTTGGTTTGG  TGGAATCCT GAACCCACTTCT  AAACACAGCCACCACCCAAGTG  ATCATCTCCTGGCTGAATGC |
| Genes  ( Mouse) |  |  |
| *Gapdh* | GCACAGTCAAGGCCGAGAAT | GCCTTCTCCATGGTGGTGAA |
| *Sox5* | CCCGTGATCCAGAGCACTTAC | CCGCAATGTGGTTTTCGCT |
| *Mmp9* | CTGGACAGCCAGACACTAAAG | CTCGCGGCAAGTCTTCAGAG |
